# *FragMAXapp*: crystallographic fragment-screening data-analysis and project-management system

**DOI:** 10.1107/S2059798321003818

**Published:** 2021-05-14

**Authors:** Gustavo M. A. Lima, Elmir Jagudin, Vladimir O. Talibov, Laila S. Benz, Costantino Marullo, Tatjana Barthel, Jan Wollenhaupt, Manfred S. Weiss, Uwe Mueller

**Affiliations:** a BioMAX, MAX IV Laboratory, Fotongatan 2, 224 84 Lund, Sweden; b Institut für Chemie und Biochemie, Freie Universität Berlin, Thielallee 63, 14195 Berlin, Germany; c Macromolecular Crystallography, Helmholtz-Zentrum Berlin, Albert-Einstein-Strasse 15, 12489 Berlin, Germany

**Keywords:** fragment screening, high-throughput data analysis, protein crystallography, drug discovery, fragment-based lead discovery

## Abstract

Crystallographic fragment screening (CFS) has become one of the major techniques for screening compounds in the early stages of drug-discovery projects. Following the advances in automation and throughput at modern macromolecular crystallography beamlines, the bottleneck for CFS has shifted from collecting data to organizing and handling the analysis of such projects. The complexity that emerges from the use of multiple methods for processing and refinement and to search for ligands requires an equally sophisticated solution to summarize the output, allowing researchers to focus on the scientific questions instead of on software technicalities. *FragMAXapp* is the fragment-screening project-management tool designed to handle CFS projects at MAX IV Laboratory. It benefits from the powerful computing infrastructure of large-scale facilities and, as a web application, it is accessible from everywhere.

## Abstract 2

The amount of data generated during crystallographic fragment-screening projects requires sophisticated automated methods to analyse the data and to identify binders. *FragMAXapp* is the MAX IV Laboratory approach to managing fragment-screening campaigns: a web application that provides scientists with access to the MAX IV computing cluster and visualization tools.

## 1. Introduction   

Fragment-based lead discovery utilizes a toolbox of biophysical methods, with X-ray crystallography-based fragment screening (CFS) being the main screening technique to obtain 3D structural information on protein–ligand complexes. In its methodological development, CFS co-evolves together with the scientific instrumentation and, more importantly, with the scientific infrastructure that supports contemporary macromolecular X-ray crystallography (MX) (Davies & Tickle, 2011 ▸; Grimes *et al.*, 2018 ▸). In its current state, CFS relies on experimental setups and data-analysis methodologies that deviate from a classical crystallographic workflow (Krojer *et al.*, 2017 ▸; Davies & Tickle, 2011 ▸; Wollenhaupt *et al.*, 2021 ▸).

Advances in MX data acquisition are setting new standards in structural biology (Grimes *et al.*, 2018 ▸). With the advent of MX beamlines at fourth-generation photon sources at synchrotron-radiation facilities such as BioMAX at MAX IV Laboratory (Ursby *et al.*, 2020 ▸), Manacá at the SIRIUS Light Source (Nascimento, 2020 ▸), AMX and FMX at NSLS II (Fuchs *et al.*, 2016 ▸) and MASSIF-1, MASSIF-2 and MASSIF-3 at ESRF–EBS (von Stetten *et al.*, 2020 ▸; McCarthy *et al.*, 2018 ▸; Svensson *et al.*, 2018 ▸), the average data-collection time has been reduced by a factor of ten to less than 40 s for a 360° ω scan. With improved beamline sample capacity allied with reliable and fast robotic sample handling (Martiel *et al.*, 2020 ▸; Ursby *et al.*, 2020 ▸; Murakami *et al.*, 2020 ▸), automated crystal centring (Ito *et al.*, 2019 ▸; Di Castro *et al.*, 2008 ▸; Schurmann *et al.*, 2019 ▸; Svensson *et al.*, 2020 ▸) and the possibility of unattended data collections (Mueller *et al.*, 2017 ▸; Cipriani *et al.*, 2012 ▸; Nurizzo *et al.*, 2016 ▸; Sanchez-Weatherby *et al.*, 2019 ▸), contemporary MX experiments generate a massive volume of data within a short time frame, for example more than 100 data sets during a single 8 h session. Finally, advances in crystal-harvesting including new tools (Barthel *et al.*., 2021 ▸) as well as new methods such as acoustic crystal harvesting (Samara *et al.*, 2018 ▸; Yin *et al.*, 2014 ▸), robot-aided harvesting (Wright *et al.*, 2021 ▸) and fully automated systems (Cipriani *et al.*, 2012 ▸) are increasing the number of crystals harvested by an order of magnitude. Thus, collecting data for CFS at such facilities is a relatively robust procedure and the main bottleneck in MX-based fragment-screening experiments is shifting from sample preparation and data acquisition to data analysis, the building of multiple models and the interpretation of the results.

Currently, automated methods for data processing and structure determination are the best way to deal with the enormous amount of data created in screening experiments. Although those methods are becoming very efficient, one major limitation is still computing power. For example, using a reasonably performing desktop computer, indexing and integrating a single high-resolution data set collected using a high-resolution detector such as an EIGER 16M can take up to 1 h 30 min. Automated and parallel approaches for data analyses benefit from exploring the solutions from several pipelines and cherry-picking the best solution, but the problem of limited computing power is aggravated. To facilitate parallel data processing for hundreds of data sets, data-collection facilities are investing in high-performance computing (HPC). Facilities such as MAX IV provide user access to their computing infrastructure, with the as-yet underestimated advantages of local availability of the data. CFS can benefit greatly from HPC-mediated data processing and analysis, as it improves the quality of the screening results and may possibly increase the sensitivity of the method.

The massive amount of raw experimental data, its processing and the refinement of all potential structures of protein–ligand complexes, the exploration of bound ligands and the corresponding ligand-binding sites, and meta analysis of the data must be treated integrally. This concept was pioneered and first implemented by Astex Therapeutics Ltd (Cambridge, UK; currently part of Astex Pharmaceuticals) within their Pyramid Platform (Davies *et al.*, 2006 ▸; Davies & Tickle, 2011 ▸). A similar logic was applied by XChem, a fragment-screening facility at Diamond Light Source, Didcot, UK that has operated since 2015. XChem users manage CFS data with a standalone graphical application called *XChemExplorer* (Krojer *et al.*, 2017 ▸), which is also available within the *CCP*4 software suite (Winn *et al.*, 2011 ▸). The application guides the user through most stages of the screening experiment, including data processing, analysis, refinement of the models and their deposition in the Protein Data Bank (Berman *et al.*, 2000 ▸). Another implementation of the concept was made by EMBL with the web-based *Crystallographic Information Management System* (*CRIMS*). This application records all experiments performed with a given target and allows automated data analysis using *Pipedream* (Vonrhein *et al.*, 2011 ▸; Smart *et al.*, 2014 ▸; Bricogne *et al.*, 2017 ▸; Kabsch, 2010 ▸; Joosten *et al.*, 2011 ▸; Collaborative Computational Project, Number 4, 1994 ▸).

**Figure 1:**
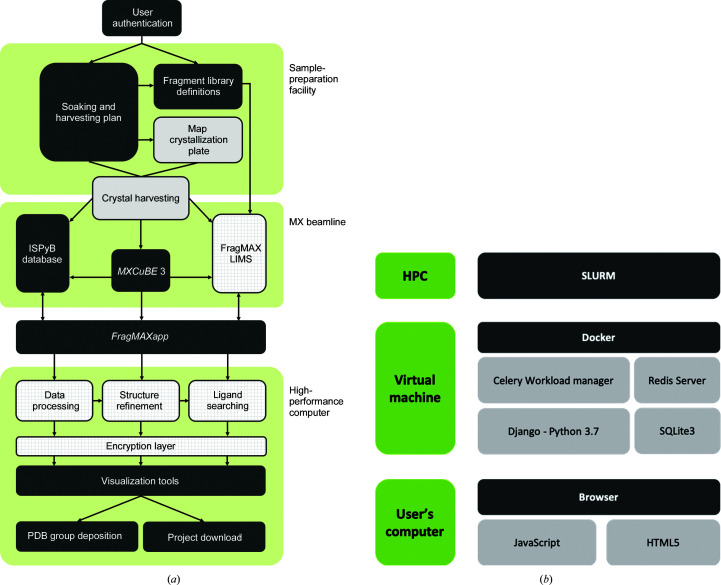
(*a*) *FragMAXapp* schematic diagram. The diagram shows connected steps and the relationship between each interaction, process and database entry. It is divided into three groups based on where the actions take place. Steps coloured dark grey use the HTML interface for interaction, light grey steps are tasks performed in the experimental laboratory and chequered steps are tasks performed in the background (for example HPC jobs). (*b*) *FragMAXapp* development design. Processing jobs are controlled by a Slurm workload manager installed in the front end of the MAX IV HPC. The three servers and the database necessary for *FragMAXapp* are deployed inside a Docker container inside a virtual machine, ensuring performance and stability of the installation. The user interface is designed using HTML5, CSS3 and JavaScript, which are available in all modern browsers.

Here, we present *FragMAXapp*, our solution to facilitate the scientific analysis, presentation and storage of large data sets from CFS and similar high-throughput crystallographic experiments (https://maxiv.lu.se/fragmax/fragmaxapp). *FragMAXapp* is a web-based expert system that links all of the steps of crystallographic ligand screening from experimental design and sample preparation to the deposition of the final structures in the Protein Data Bank. The application focuses on user accessibility, flexibility of the data analysis and high performance, linking the MAX IV HPC infrastructure with a selection of automated data-processing and analysis workflows (Fig. 1 ▸ *a*). *FragMAXapp* was designed within the operation framework of the BioMAX beamline (Ursby *et al.*, 2020 ▸) and connects to other beamline applications, such as the ISPyB sample database (Beteva *et al.*, 2006 ▸) and the *MXCuBE* experiment-control software (Mueller *et al.*, 2017 ▸; Oscarsson *et al.*, 2019 ▸). However, the abstraction level of *FragMAXapp* provides modularity that allows it to be ported and adapted to other sites. In fact, at the time of this publication, working versions of *FragMAXapp* are deployed on BESSY II at the Helmholtz-Zentrum Berlin (Mueller *et al.*, 2015 ▸; Wollenhaupt *et al.*, 2021 ▸) and at the University of São Paulo using data from the SIRIUS Light Source (Noske *et al.*, 2021 ▸).

## 2. Software design and operation   

*FragMAXapp* has been developed using Python3/Django (Django version 2.2.1; https://djangoproject.com) as its back end and using JavaScript for front-end features (Fig. 1 ▸ *b*). The project definition, including protein models, the structures of the small molecules and user information, is stored in an SQLite3 database.

As a web application, *FragMAXapp* requires a web browser with the support of WebGL (Web Graphics Library API, Mozilla). This feature allows data analysis to be performed using virtually any device connected to the internet. The user interface is designed using HTML5, CSS3 and JavaScript for responsiveness and functionality. User access can be obtained using the ISPyB authentication system, which provides access to data and results based on the MAX IV user-account system, or with a local account with access privileges granted by the system administrator.

The application offers a variety of choices for data processing, including *DIALS* (Winter *et al.*, 2018 ▸) and *XDS *(Kabsch, 2010 ▸) through *xia*2 (Winter *et al.*, 2018 ▸; Evans, 2006 ▸; Gildea *et al.*, 2011 ▸),* autoPROC* (Vonrhein *et al.*, 2011 ▸), *XDSAPP* (Krug *et al.*, 2012 ▸; Sparta *et al.*, 2016 ▸), *fastdp* (Winter & McAuley, 2011 ▸) and *EDNAproc* (Incardona *et al.*, 2009 ▸). Automated structure refinement is performed by *DIMPLE*, *BUSTER* (Bricogne *et al.*, 2017 ▸; Smart *et al.*, 2012 ▸) and *fspipeline* (Schiebel *et al.*, 2016 ▸). The ligand-finding step is performed by *Rhofit* (Smart *et al.*, 2014 ▸), *Phenix LigandFit* (Terwilliger *et al.*, 2006 ▸, 2007 ▸) and *PanDDA* (Pearce *et al.*, 2017 ▸), with ligand coordinates and restraints generated by *Phenix eLBOW* (Moriarty *et al.*, 2009 ▸), *GRADE* (Smart *et al.*, 2011 ▸) or *AceDRG* (Long *et al.*, 2017 ▸). Within the application interface, structural representation of the ligands is created using the *RDKit* (Landrum, 2012 ▸) and 3*Dmol* (Rego & Koes, 2015 ▸) libraries. Protein atomic coordinates, electron-density maps and reciprocal space are displayed using the *UglyMol* (Wojdyr, 2016 ▸) library with minor modifications that allow the visualization and comparison of multiple models. Interactive plots for data statistics are created using the *D*3 library (Bostock, 2017 ▸). The X-ray diffraction representations are created using *Adxv* (Arvai, 2012 ▸) with sufficient images to cover 1° of crystal oscillation. The selection of software available in a specific facility is defined during the deployment of the application.

The MAX IV HPC infrastructure manages its workload using Slurm, which is interfaced with the *FragMAXapp* server using a Celery workload manager and a Redis server. These two services run together with the Django server to enable promise-like requests for data while maintaining fluid navigation in the web app.

All services run from a Docker container allocated in a virtual machine configured with GitHub’s continuous integration feature, allowing easy deployment and updating of the tools. The Docker deployment uses NGINX/uWSGI to serve the application.

### 2.1. Project management   

The application organizes experimental data and their analysis into application-specific projects. A *FragMAXapp* project is defined by a unique combination of four parameters: the protein name, a common identifier of the compounds (for example the name of the fragment library), the BioMAX proposal number and the corresponding data-collection sessions (Supplementary Fig. S1). These values can be updated to incorporate follow-up experiments related to the same protein within the same proposal.

To facilitate work with sensitive information and to allow users to follow up specific policies for data handling, the project can be created in an encrypted mode. With this option the project-related data are encrypted, and are decrypted only temporarily when the user or the processing software require access. For increased security, the user can manage the availability of the encryption key. It is possible to download, remove and re-upload the encryption key that is used for the project. When the encryption key is removed, the project data effectively become inaccessible unless the user re-uploads the key.

**Figure 2:**
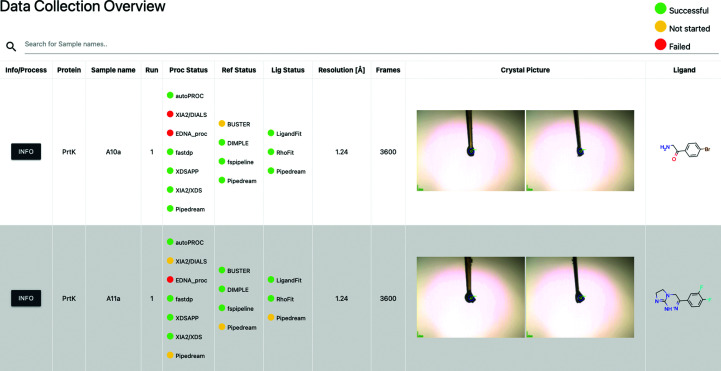
*FragMAXapp* Project Overview page. It displays information about the data collection and the status of data processing. A full representation of the user interface is available in Supplementary Fig. S2.

Beyond meta-management of the projects, *FragMAXapp* allows the examination of individual data sets and other experimental details. Often, this information is stored in sample databases such as ISPyB and can be retrieved at any time by the user. With *FragMAXapp*, browsing this information is also possible (Fig. 2 ▸).

### 2.2. Sample management   

**Figure 3:**
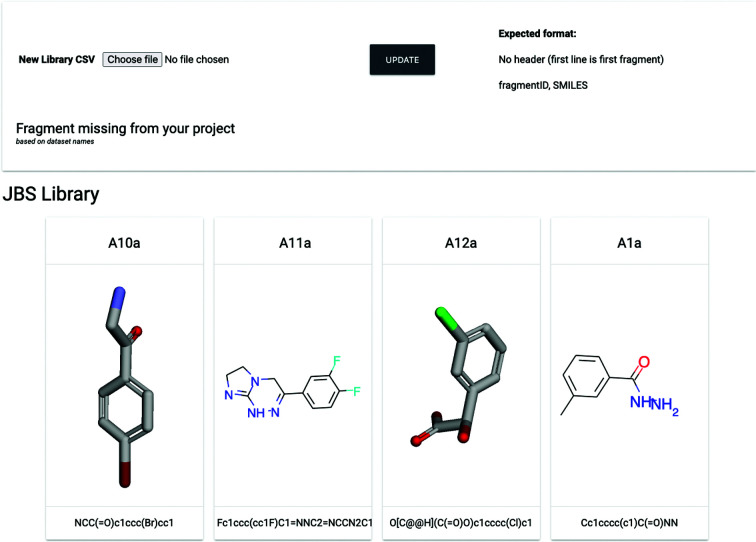
*FragMAXapp* sample-management page. The 2D representations of the ligands are generated using *RDKit*, while 3D representations are displayed using the 3*Dmol* library. This page allows additions or updates to the sample definitions within the project. A full representation of the user interface is available in Supplementary Fig. S3.

Definition of the screening collection is an essential step in project management. *FragMAXapp* requires the user to upload a comma-separated file (CSV) with library specifications, including compound identifiers, which must be identical to the sample names, and optional SMILES strings for the chemical structures of the ligands. The library view page displays updated information about the sample database, with 2D and/or 3D representations of each molecule, and the SMILES code for each compound ID in the project (Fig. 3 ▸). Missing entries in the sample database are displayed in the page header, based on the sample names available and the compound IDs in the database. It is possible to update the definition of the samples, which allows work with follow-up experiments within the same project.

### 2.3. Data processing and structure solution   

**Figure 4:**
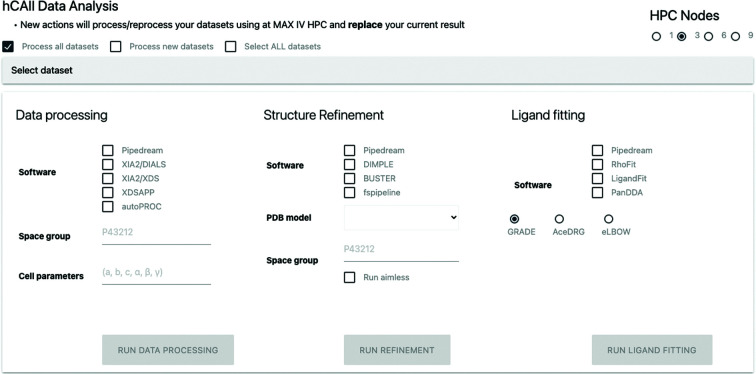
*FragMAXapp* Data Analysis page. An extensive selection of software and pipelines is available to analyse the data. A full representation of the user interface is available in Supplementary Fig. S4.

The most powerful feature of *FragMAXapp* is its capability to handle numerous data-analysis methods in a parallel and multiplexed manner (Fig. 4 ▸). It is widely accepted that no single combination of processing and analysis software outperforms any available solution, meaning that only after testing all possible combinations of automated pipelines is it possible to identify the best strategy for a particular data set (Powell, 2017 ▸). As we focus on fully automated data-analysis schemes, *FragMAXapp* offers a combination of six data-processing software packages: *autoPROC*, *xia*2/*DIALS*, *xia*2/*XDS*, *EDNAproc*, *fastdp* and *XDSAPP*, three structure-refinement pipelines, *BUSTER*, *DIMPLE* and *fspipeline*, and three automatic ligand-searching programs, *Phenix LigandFit*, *Rhofit* and *PanDDA*. Selecting all available software can generate a total of 54 combinations for the evaluation of each diffraction data set. For each software, *FragMAXapp* suggests options to optimize its function, such as the definition of resolution cutoffs for the data, alternative frame ranges for the data sets, the application of Friedel’s law during processing, the water-placement method in structure-refinement stages and many more, efficiently acting as a unified GUI front end. Providing customized parameters for data analyses is optionally possible; otherwise the application chooses default values when no input is given. Using a built-in selection table, only selected data sets can be analysed or re-analysed. To ensure that all software will have the necessary information to run properly, *FragMAXapp* keeps a set of mandatory inputs.

A dedicated tab is available to check all currently running data-analysis jobs and inspect the processing logs. This feature displays live information from the computing nodes and the queueing system. Within this page, it is possible to remove jobs from the processing queue or to kill running analyses.

*FragMAXapp* integrates *PanDDA* analysis, with several features that allow the sensitivity of the screening experiment to be increased. Under the *PanDDA* tab on the Data Analysis page, it is possible to choose which data-analysis output will be used to run *PanDDA*. If the result is missing, *FragMAXapp* will check whether a solution was offered by other combinations of analysis software. Alternatively, the application offers the *FragPLEX* option, a method to decide which results to submit for *PanDDA* analysis. The method is based on the evaluation of processing and refinement statistics. The first step of the selection process discards all processed data sets with an overall *R*_meas_ of >10%. The second step ranks solutions by higher ISa value and resolution and lower *R*_free_/*R*_work_. The selection is saved in the *FragMAXapp* database. In our tests, applying this selection logic allows the retrieval of all hits found by combining hits from all individual analysis (*i.e.* a single combination of data processing and structure refinement). Additionally, *FragMAXapp* offers the option to build ground-state models using exclusively known apo structures or to use data sets without peaks in the *Z*-maps (the latter requires at least one *PanDDA* analysis before use). In the final steps of *PanDDA* analysis, the evaluation of modelled ligands can be performed through the *PanDDA* Giant scoring scripts (Pearce *et al.*, 2017 ▸), and the radar plots of fitting quality generated in this step are shown in the web app.

### 2.4. Data visualization   

**Figure 5:**
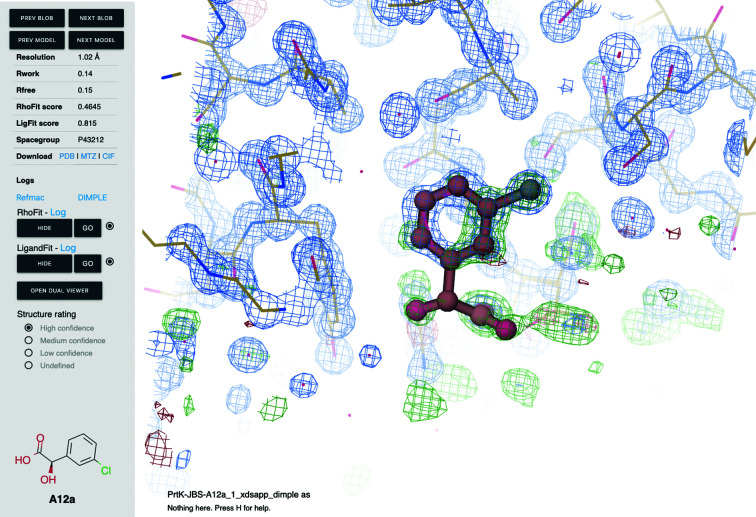
*FragMAXapp* density viewer. The viewer features unexplained blobs and navigation between the models, information about the result, log access, ligand navigation for automatic ligand-fitting results and structure scores. The density viewer is based on *UglyMOL*. A full representation of the user interface is available in Supplementary Fig. S5.

The results generated by the automatic analysis pipelines can be visualized in the web browser without any external tools or plugins. It is possible to open and read the output logs from each software used, visualize reciprocal-lattice representation and load refined models with electron density with a *Coot*-like look and feel using *UglyMol* (Fig. 5 ▸). Besides, the web app provides interactive and comprehensive overview plots for many statistical parameters such as resolution, ISa, *R*_work_/*R*_free_ and unit-cell parameters. This allows the whole CFS data evaluation to be compared within one view, including averaged values and standard deviations for various combinations of methods. These plots can be extremely helpful to obtain a quick overview of the data analysis and to identify data sets that require reprocessing or manual examination.

The *PanDDA* results visualization within *FragMAXapp* displays the event map and the average map obtained from the ground-state model side by side (Supplementary Fig. S6). Using this visual feature, it is easier to interpret unexplained or modelled densities that are also present in the averaged ligand-free model.

### 2.5. Export tools   

After the analyses are finished, the web app provides tools to download the data. A variety of choices for which data to export is available, combining process data, log files, structure files and electron densities. Pre-processing results using *PDB-REDO* (Joosten *et al.*, 2012 ▸) before deposition is optional but is highly recommended as it improves the quality of automatically refined structures and prepares them for subsequent deposition in the PDB. Using this feature, users are no longer required to manually deposit every model; instead, a compressed archive file with structures, reflection data and the index file is generated, ready to be uploaded using the OneDep system. The compressed file can be downloaded from *FragMAXapp* or using regular tools available at MAX IV. At the time of publication, SFTP and Globus (Foster, 2011 ▸) are offered to users. A final option supported by *FragMAXapp* is uploading of the raw data to public diffraction databases such as proteindiffraction.org (Grabowski *et al.*, 2016 ▸).

## 3. Fragment screening using *FragMAXapp*   

To test our implementation of the automated methods, a fragment-screening campaign was performed using proteinase K (PROK) from *Tritirachium album* (UniProt accession code P06873). PROK is a study model of serine proteases (Larson *et al.*, 2009 ▸). The materials and methods used to obtain PROK crystals, including the soaking and harvesting procedures, are described in Section S1. Data-collection parameters are given in Table 1 ▸.

**Figure 6:**
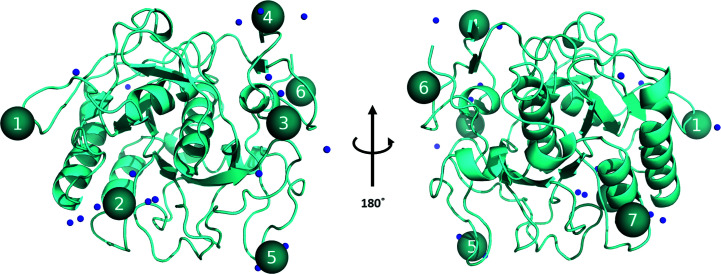
Proteinase K sites numbered from 1 to 7 as identified by *PanDDA* analysis.

**Figure 7:**
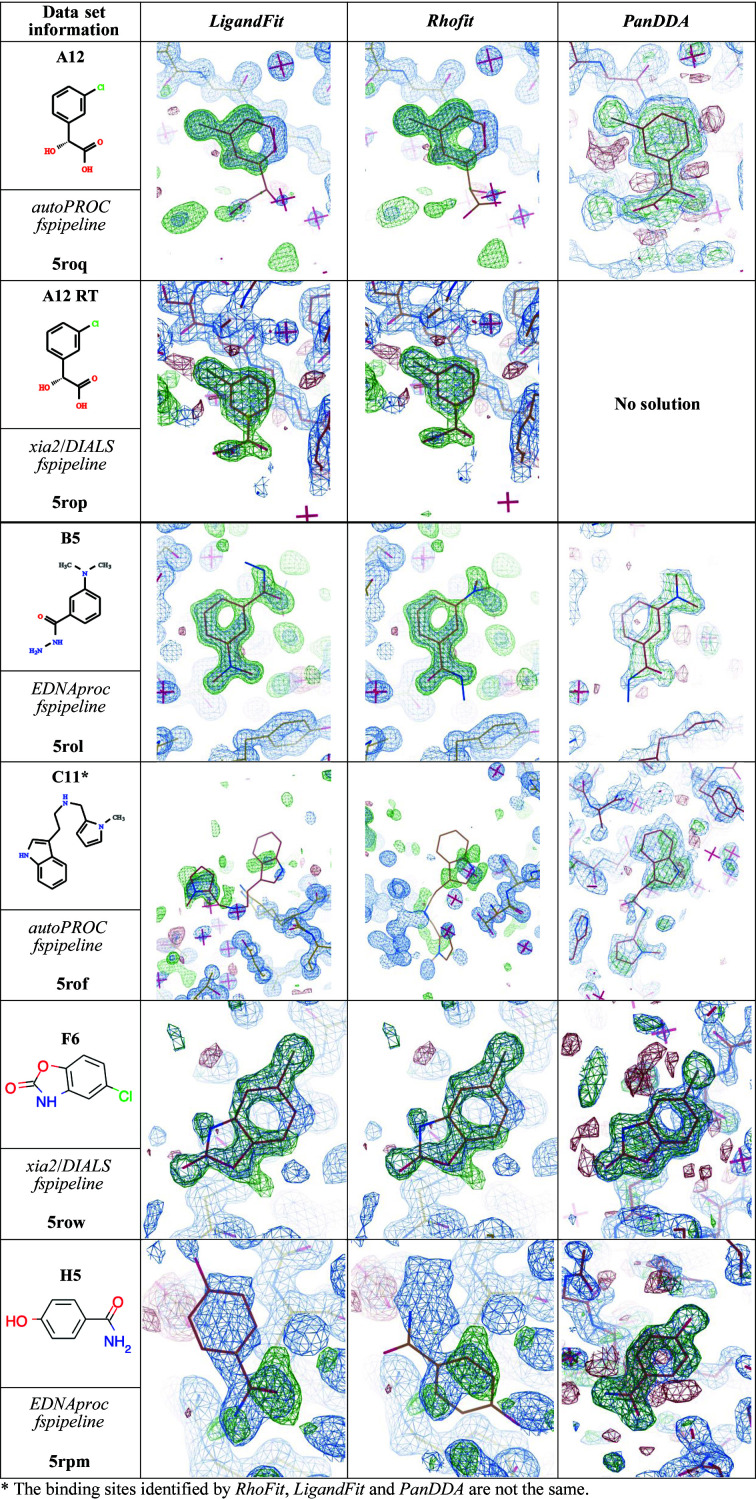
Automated ligand-searching comparison. The *LigandFit* and *Rhofit* columns take the highest scored ligand from each method. The *PanDDA* column shows ligands that were manually modelled. Ligands with a different binding site for different methods are annotated. All structures and density maps are publicly available.

Fragment screening of the 96 samples prepared as ligand soaks using the Frag Xtal Screen library, and an extra 40 apo crystals, was performed and data analysis was performed using *FragMAXapp*. The plots, with an overview of data processing and structure refinement, are available in Supplementary Table S1. Applying different automated ligand-fitting (*LigandFit* and *Rhofit*) and ligand-searching (*PanDDA* with manual inspection of *Z*-maps) methods led to very time-efficient hit identification in two weeks of data analysis, including post-refinement and preparation for deposition. This is remarkable when compared with the expected time required to perform the analysis of 136 data sets. At the time of writing this article (GitHub commit https://github.com/FragMAX/FragMAXapp/commit/14199af229a43b25a5994c5d58bc92df523cb31d), ligand modelling requires *Coot* (Emsley *et al.*, 2010 ▸), which can be achieved by accessing a virtual computer at synchrotron facility. *PanDDA* analysis was performed by allowing *FragMAXapp* to select the best combination of data processing and structure refinement; the selection is available in Section S1. *PanDDA* identified events and clustered them into seven sites (Fig. 6 ▸), which were compared with standard difference density map analysis and automated ligand-fitting methods (Fig. 7 ▸). In general, when the unexplained density was sufficiently defined to fit a large portion of the corresponding fragment, all methods modelled the bound ligand with a certain degree of precision and would require little real-space refinement to make it ready for deposition. However, using the information from the *PanDDA* event map is likely to improve the model, as shown for fragments A12 and B5 in Fig. 7 ▸. In several cases, such as fragments B7 and C11, automated fitting methods disagree on the site in which to locate the ligand, making the *PanDDA* event map decisive in ligand modelling. To highlight how each of these methods compares with each other and what the user can typically expect from the automated methods in *FragMAXapp*, no further modelling was performed on the protein–ligand complexes. Overall, *PanDDA* led to an increase in the hit rate, from ten to 18 identified binding events, and a better modelling of bound fragments with partial occupancy.

The results from the PROK fragment screening and the apo structures used to build the ground-state model in *PanDDA* were submitted to the Protein Data Bank (PDB) using the export tool built into* FragMAXapp*. Overall, this fragment-screening campaign is an example of the application of the software and its capabilities, demonstrating what the user can expect from the web app. The fragment-screening platform of MAX IV, including *FragMAXapp*, has also been used for the validation of a structurally diverse fragment library collection, F2X-Entry, on two protein targets (Wollenhaupt *et al.*, 2020 ▸).

## 4. Conclusions   

Crystallographic fragment screening is becoming a common method in the early stages of drug-discovery projects, sometimes replacing other biophysical methods as the primary screening technique. Advances in light-source facilities have shifted the bottleneck from generating data to analysing data, requiring equal efforts to improve software support to enable CFS. The strong automation provided at modern MX beamlines, not only for data collection but also for data processing, enables medicinal chemistry-focused groups to utilize structural biology within their research.

*FragMAXapp* was developed with a focus on providing the necessary tools to manage, process, analyse and export fragment-screening projects from a web browser. The host facility benefits from software that is easy to deploy and develop, based on popular programming languages such as Python and JavaScript. With our plugin-based system, adding or removing functionalities is possible, thus adapting *FragMAXapp* to a variety of scenarios. The user will benefit from a zero-installation setup that is ‘ready to go’ from any internet-connected device. Furthermore, remote data analysis significantly decreases the time required to obtain results by skipping data transfer and using highly parallelized computing architecture. Similar solutions available at other sites such as *XChemExplorer* and *CRIMS* lack the extensive data-analysis features and/or easy access to the application through a web browser. Therefore, *FragMAXapp* will set the standard for CFS project-management and data-processing tools with its full web-stack implementation, parallel computing support and user-friendly interface.

## 5. Availability   

Documentation and source code for *FragMAXapp* can be obtained from the FragMAX project GitHub page (https://github.com/FragMAX/FragMAXapp) free of charge for academic use or noncommercial applications. Detailed tutorial and other instructions can be found on the *FragMAXapp* project page (https://fragmax.github.io/).

## Supplementary Material

Supplementary Figures, proteinase K fragment screening and Supplementary Table. DOI: 10.1107/S2059798321003818/tz5106sup1.pdf

## Figures and Tables

**Table 1:** Data-collection parameters for PROK crystals at BioMAX

	Cryo temperature	Room temperature
Wavelength (Å)	0.976	0.976
No. of images	3600	1800
Oscillation (°)	0.1	0.20 (helical scan)
Exposure (ms)	11	10
Photon flux (photons s^−1^)	∼1 × 10^12^	∼1 × 10^11^
Beam size (µm)	50 × 50	50 × 50
Resolution (at the edge) (Å)	1.24	1.24
