# Supplementary material for: FragMAXapp: crystallographic fragment-screening data-analysis and project-management system
Source: Acta Crystallogr D Struct Biol. 2021 May 14;77(Pt 6):799–808. doi: 10.1107/S2059798321003818 (PMC8171072; doi:10.1107/S2059798321003818)
Supplement: Supplementary file 1 [file d-77-00799-sup1.pdf]

STRUCTURAL
BIOLOGY

Volume 77 (2021)

Supporting information for article:

FragMAXapp: crystallographic fragment screening data analysis and project management system

Gustavo M. A. Lima, Elmir Jagudin, Vladimir O. Talibov, Laila S. Benz, Marullo Costantino, Tatjana Barthel, Jan Wollenhaupt, Manfred S. Weiss and Uwe Mueller

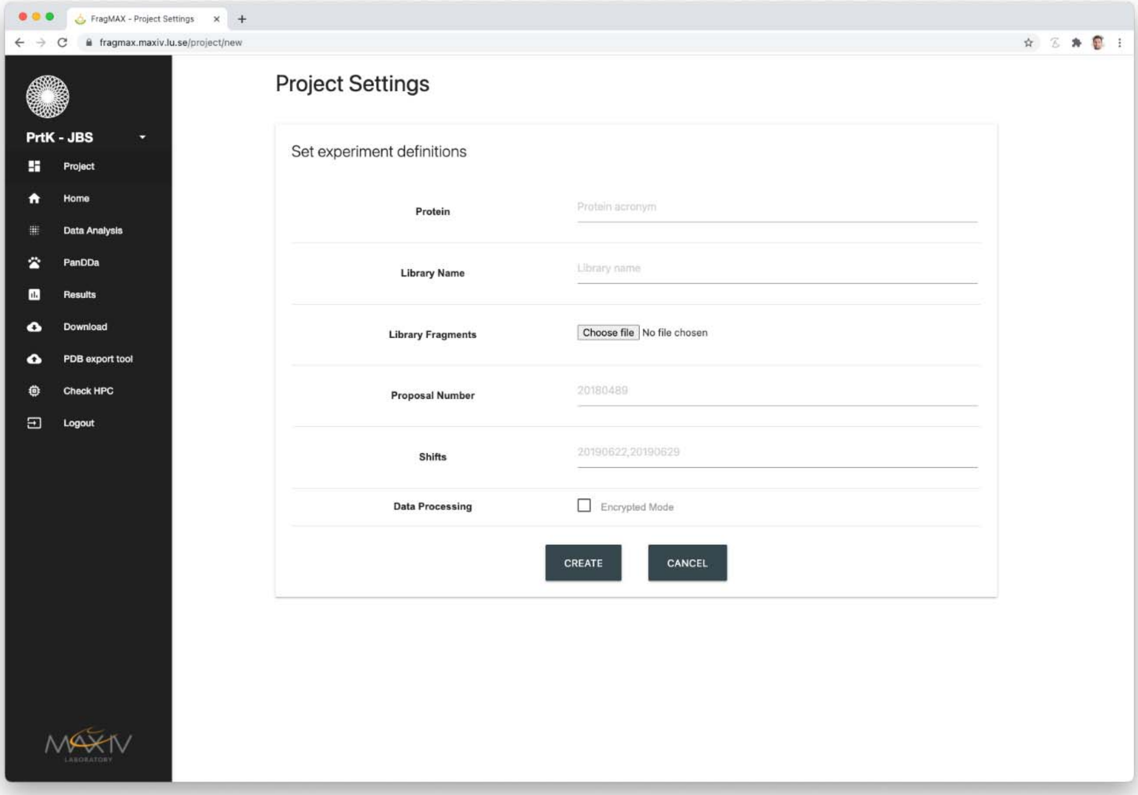

Figure S1 Project Setting page. The minimum required information is validated before project creation.

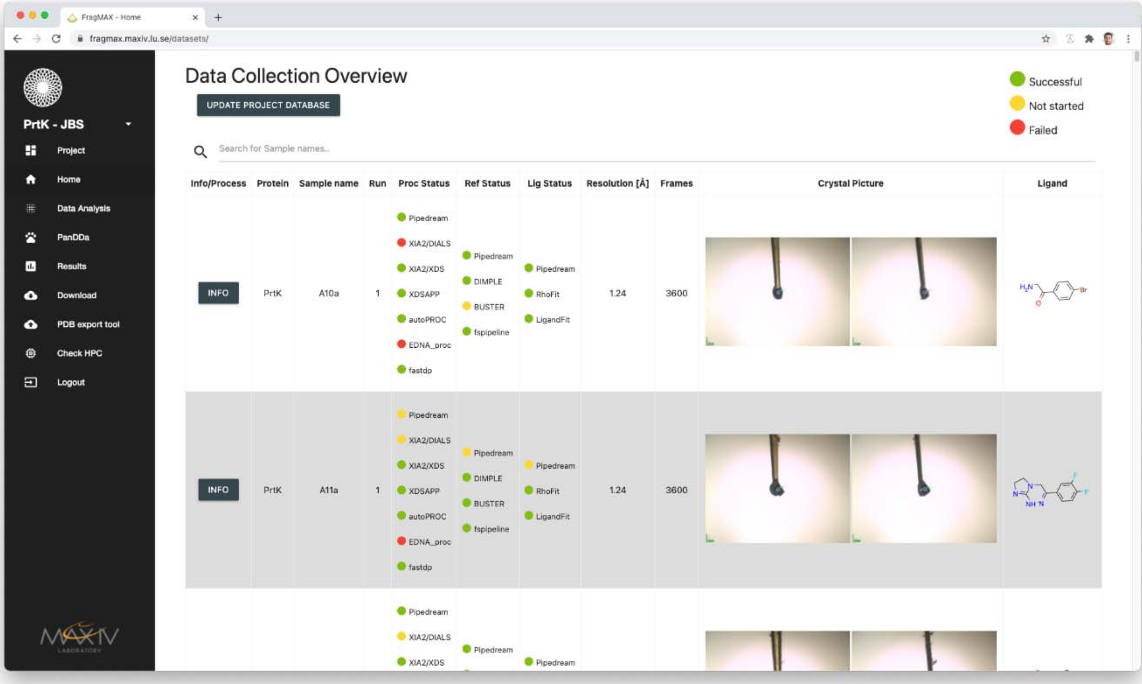

Figure S2 Fragment screening project Home Page, displaying information about the data collection parameters, the crystal, fragment and data processing progress.

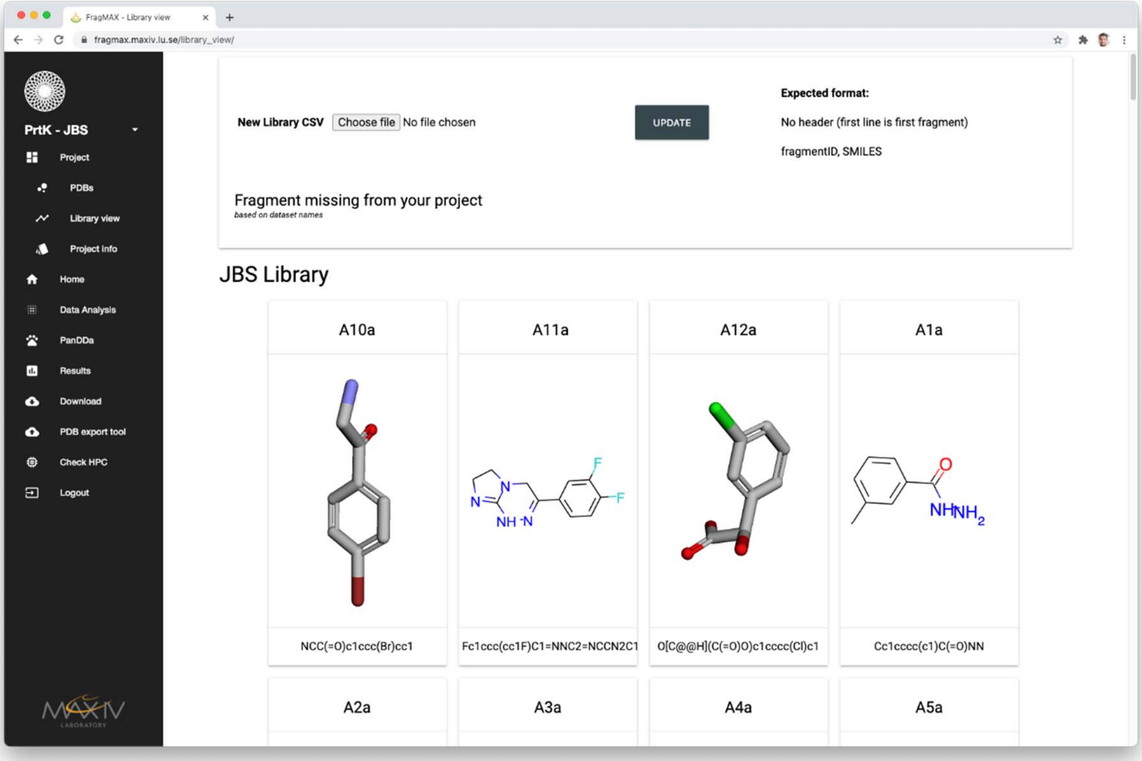

Figure S3 Full reproduction of sample management page interface.

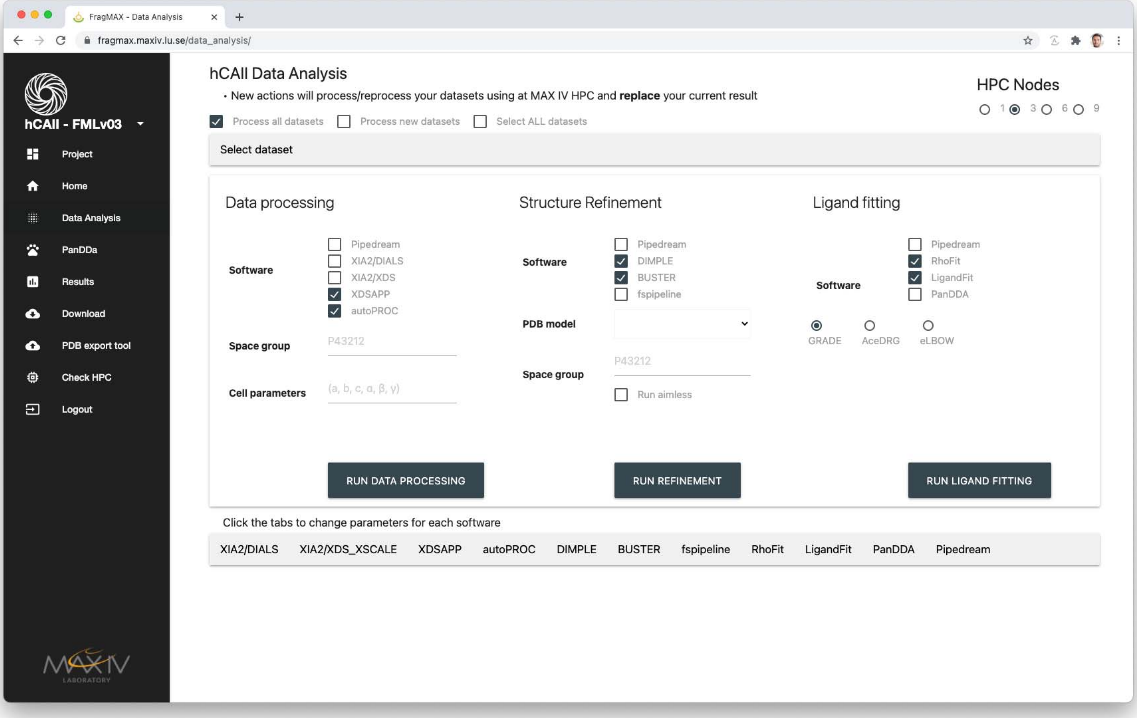

Figure S4 Full reproduction Data Analysis interface. In this view, the submission buttons are enabled due to the selection of pipelines.

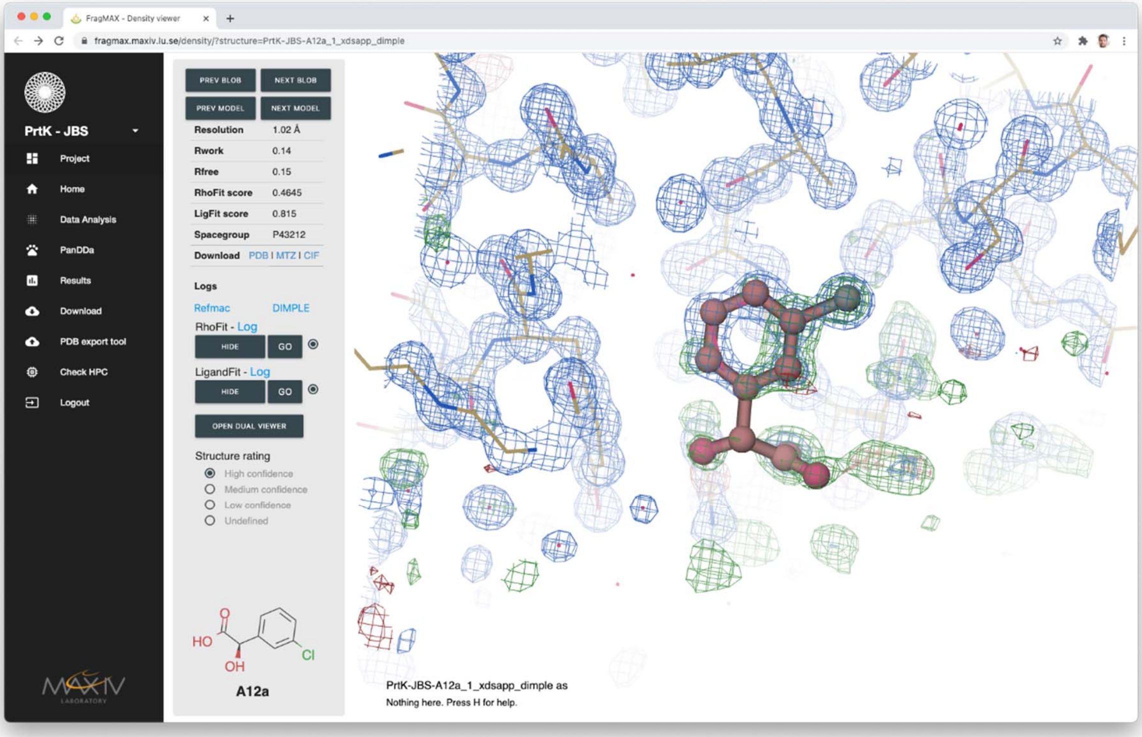

Figure S5 Full reproduction of Results view.

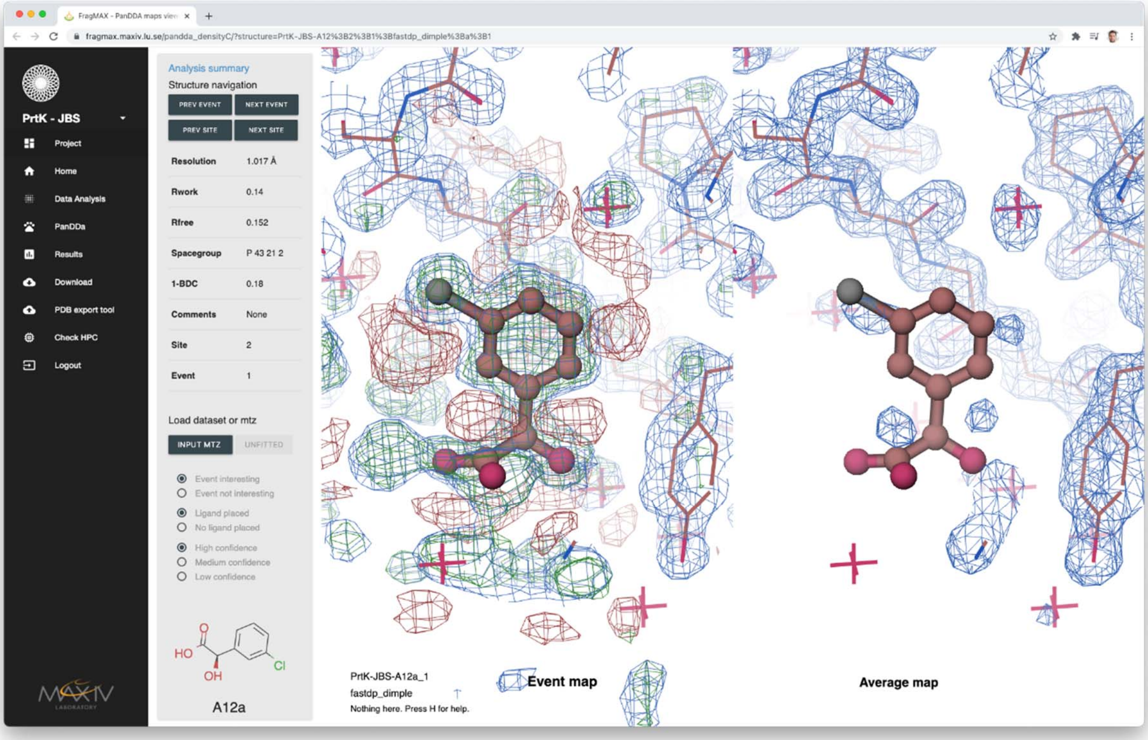

Figure S6 PanDDA events viewer. The side-by-side comparison between the event and average maps from PanDDA help identify false-positive events during the analysis. From the webapp, it is

possible to see information about the event and the user-annotated confidence level. The navigation function moves the view to different site centromeres.

S1. Proteinase K fragment screening

S1.1. Material and Methods

The protein was purchased (Jena Bioscience, Germany) and crystallised using sitting drop method as described in previous work (Larson *et al.*, 2009) on MRC3 plates (SWISSCI, Switzerland) using a Mosquito liquid handling system (TTP Labtech, UK). The fragment library Frag Xtal Screen was purchased (Jena BioScience, Germany) and handled according to the manual instructions. For this experiment, we renamed the fragments from its original convention (1 to 96) to an alphanumeric convention equivalent to its position in the 96-well storage plate (A1 to H12). The crystallisation drops were 300 nL containing a mixture of 150 nL of precipitating solution (1.2 M Ammonium Sulfate, 0.1 M Tris-HCl, pH 8.0) and 150 nL of 10 mg.mL^{-1} Proteinase K solution. Suitable single crystals were obtained after 48 h, with sizes ranging from 50 to 100 µm. The crystals were soaked for 2 hours using 300 nL at 50 nM of each fragment dissolved in a precipitant solution containing 20% DMSO, for a final DMSO concentration of 10% after addition to the crystallisation drop. To transfer the fragment solutions on top of the crystallisation drop, the Crystal Shifter (Oxford Labtec, UK) was used with the crystal plate in one of its plate holders and the solubilised fragment plate in the second one. After soaking, crystals were harvested with Crystal Shifter assistance and cryo-cooled in LN2. Diffraction data of the identified hits from the cryo-experiment were also collected at room temperature (RT). The samples for RT experiment were prepared using the same protocol described in this section, mounting the crystal loops inside MicroRT capillaries (MiTeGen, USA) instead of cryo-cooling in LN2. Data collection was performed at BioMAX beamline, with experimental parameters specified in Table 1.

S1.2. Results

Using FragPLEX selection and PanDDA to analyse the data, 18 fragments out of 96 were found, with an 18,75% hit rate (Table S1).

Table S1 Complete automated ligand searching comparison. Every hit found using PanDDA is compared to other automated methods. The dataset information column displays the fragment used during soaking, the methods used to process and refine the dataset, and the PDB ID of the final structure.

Dataset Information	LigandFit	RhoFit	PanDDA
**A11*** 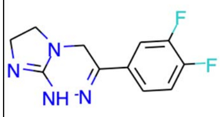	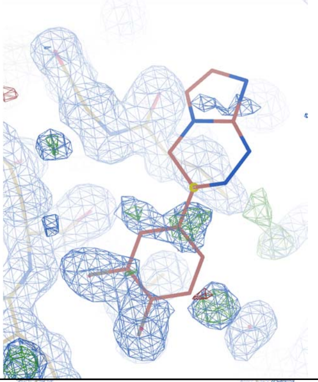	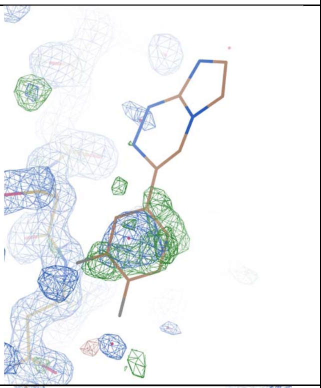	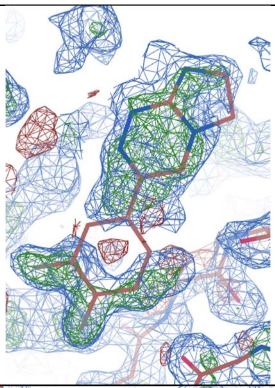
fastdp<br>fspipeline<br>**5RPH**			
**A12** 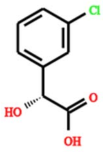	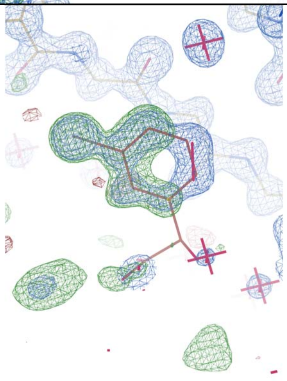	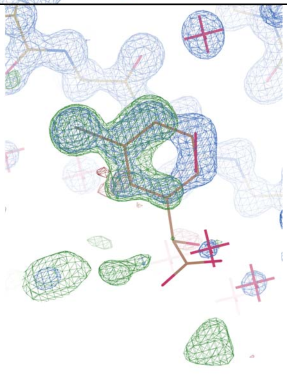	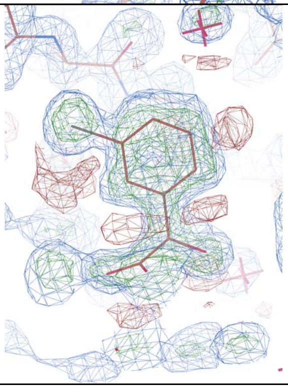
autoPROC<br>fspipeline<br>**5ROQ**			
**A12 RT** 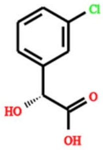	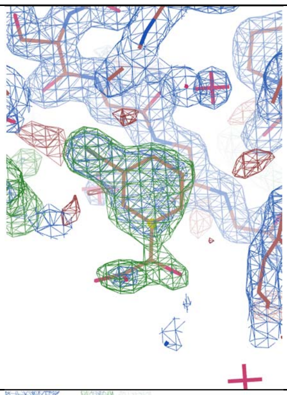	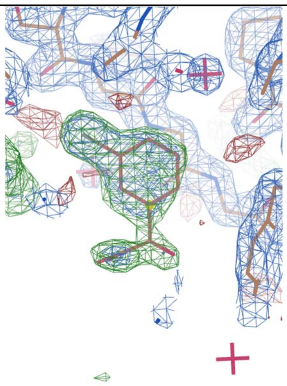	**No Solution**
xia2/DIALS<br>fspipeline<br>**5ROP**			
**B5** 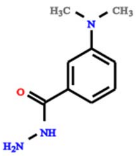	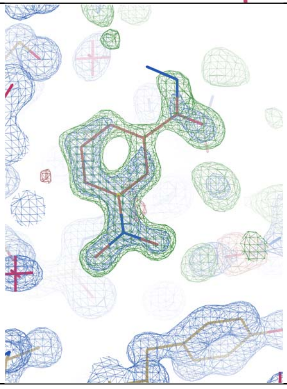	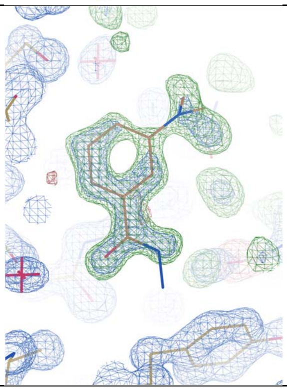	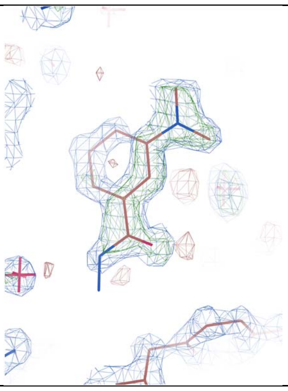
EDNA_proc<br>fspipeline<br>**5ROL**			
**B7** 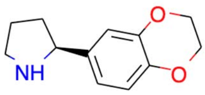			

fastdp<br>fspipeline<br>**5RP9**	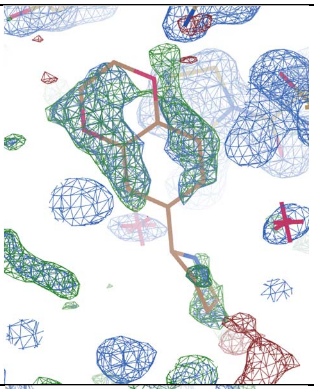	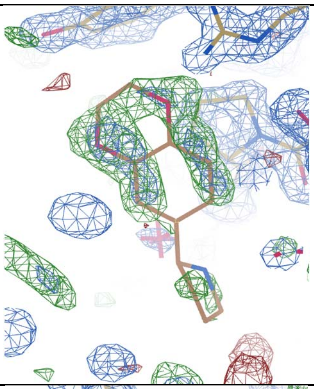	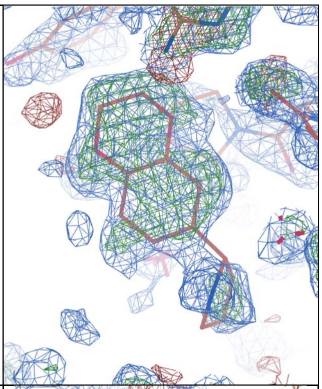
**B9**<br>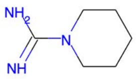<br>xia2/XDS<br>DIMPLE<br>**5RPK**	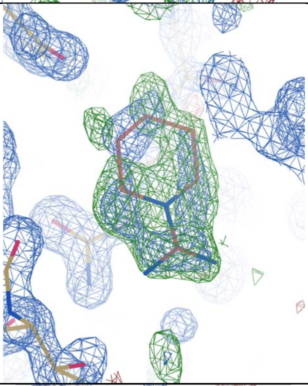	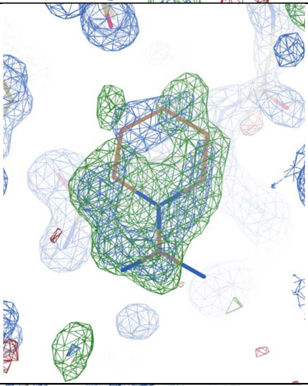	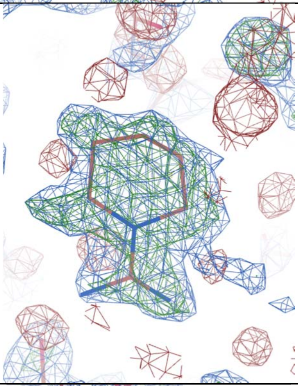
**B12**<br>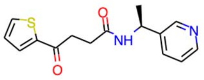<br>fastdp<br>fspipeline<br>**5RPJ**	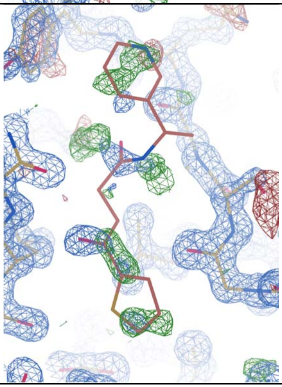	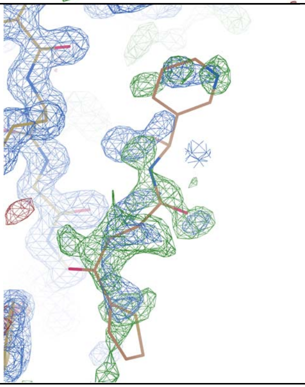	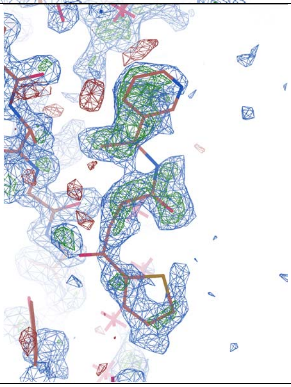
**C2**<br>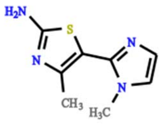<br>fastdp<br>fspipeline<br>**5RP6**	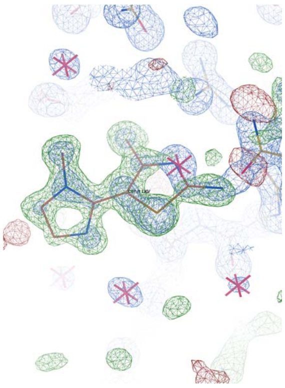	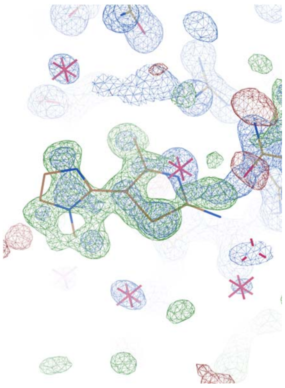	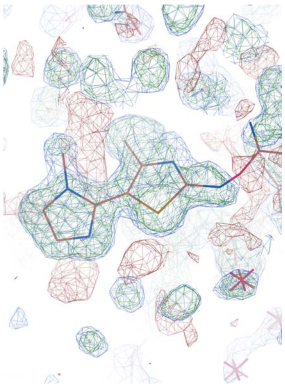
**C8**<br>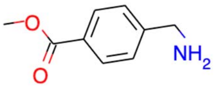	**No solution**	**No solution**	

autoPROC<br>fspipeline<br><br>**5RPL**			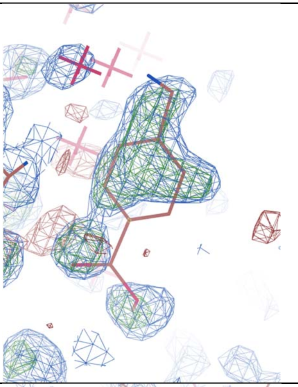
**C11***<br>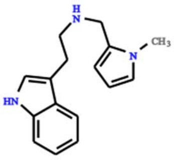<br>autoPROC<br>fspipeline<br><br>**5ROF**	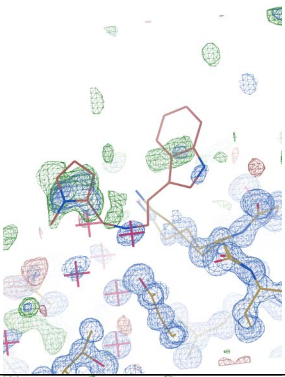	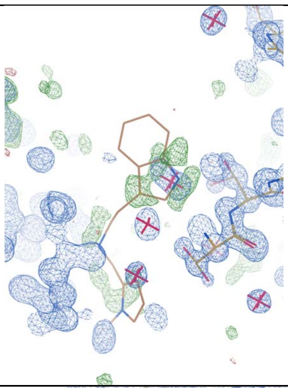	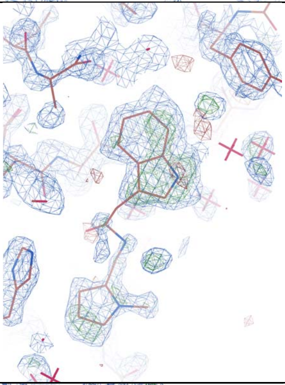
**D12**<br>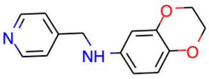<br>xia2/DIALS<br>fspipeline<br><br>**5RP7**	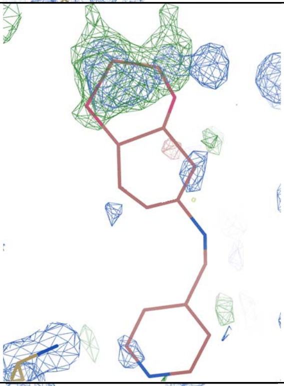	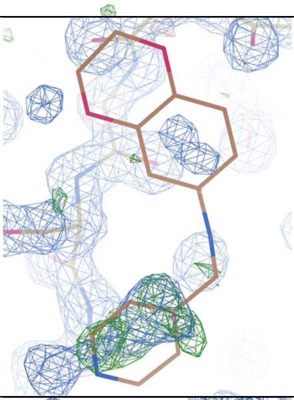	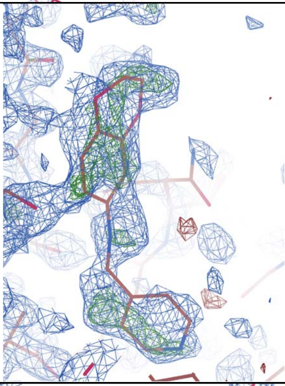
**E3***<br>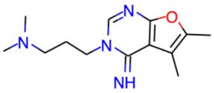<br>autoPROC<br>fspipeline<br><br>**5RPA**	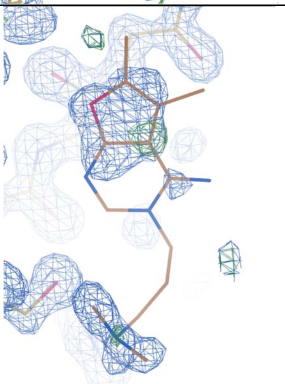	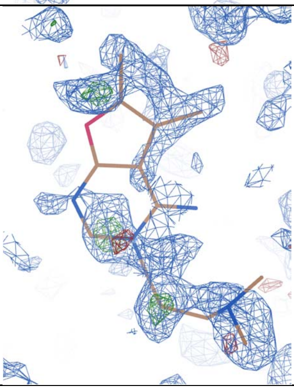	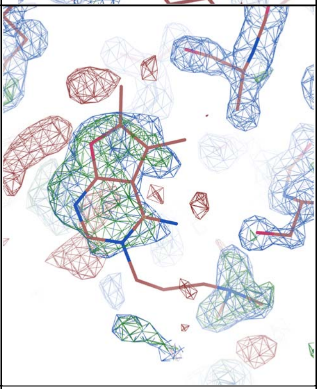
**E4**<br>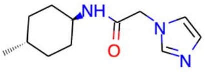	**No Solution**	**No Solution**	

xia2/DIALS<br>fspipeline<br><br>**5RON**			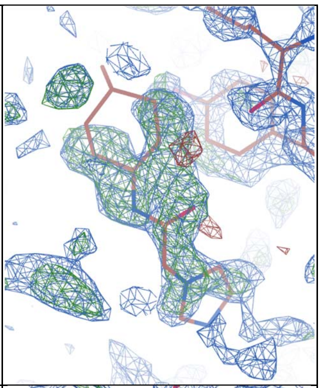
**F1**<br>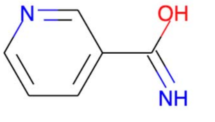<br>XDSAPP<br>fspipeline<br><br>**5ROR**	**No solution**	**No solution**	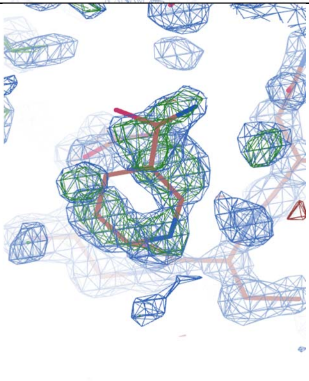
**F6**<br>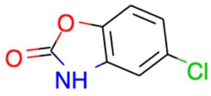<br>xia2/DIALS<br>fspipeline<br><br>**5ROW**	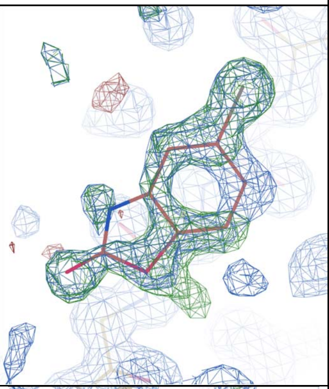	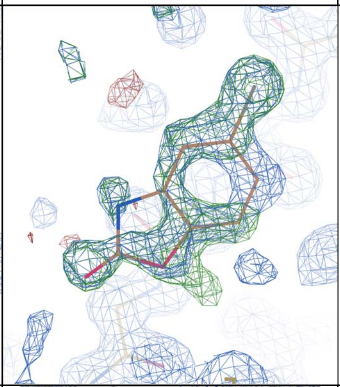	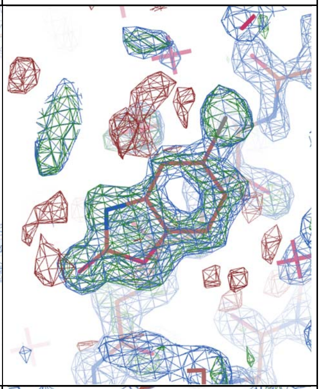
**F12***<br>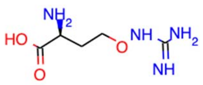<br>xia2/DIALS<br>BUSTER<br><br>**5RPD**	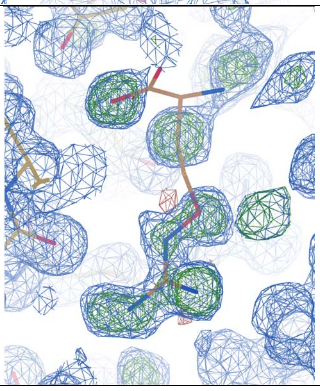	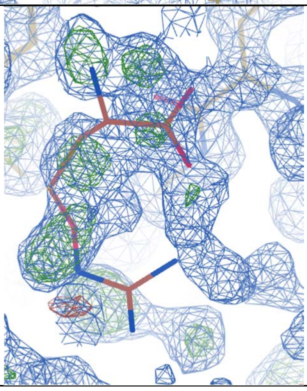	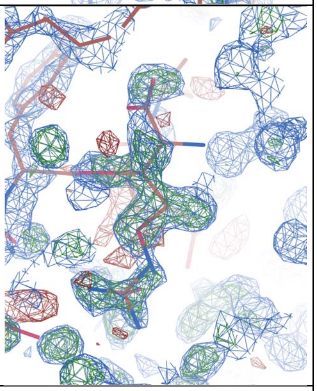
**H2**<br>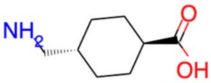		**No solution**	

XDSAPP<br>fspipeline<br><br>**5RPG**	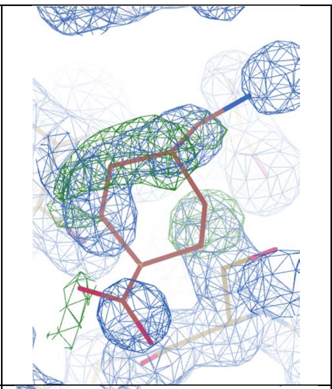		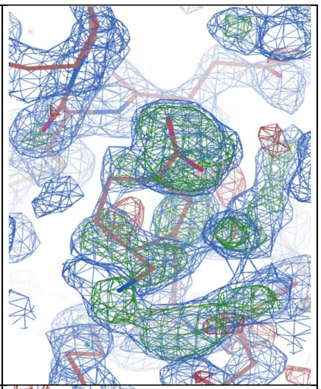
**H3***<br>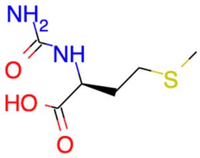<br>autoPROC<br>fspipeline<br><br>**5RPC**	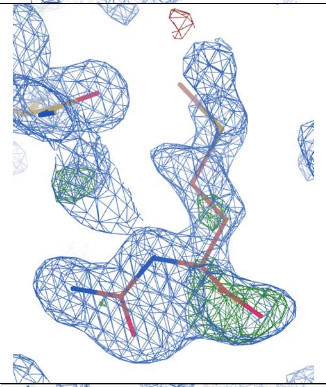	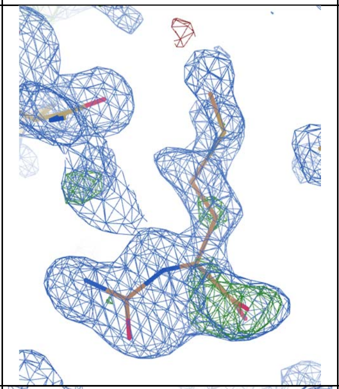	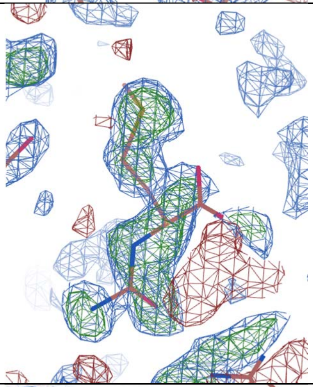
**H5**<br>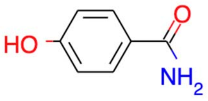<br>EDNA_proc<br>fspipeline<br><br>**5RPM**	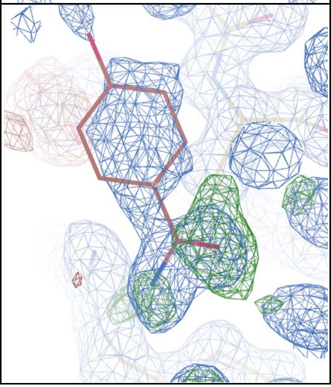	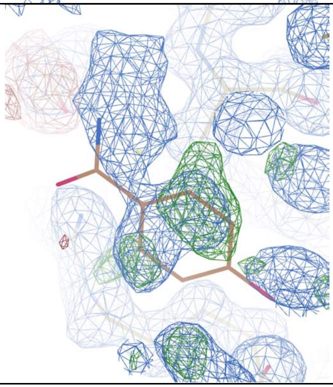	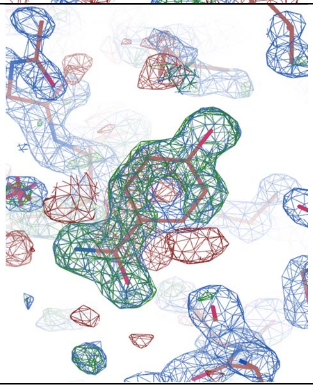

* The binding site identified by RhoFit, LigandFit and PanDDA is not the same.
